# Stakeholder priorities for ART initiation and early retention strategies in Malawi: a qualitative study comparing international and national perspectives

**DOI:** 10.1186/s12889-025-23484-8

**Published:** 2025-07-05

**Authors:** Santhi Hariprasad, Khumbo Phiri, Marguerite Thorp, Katherine Holland, Rose Nyirenda, Sundeep Gupta, Sam Phiri, Lora L. Sabin, Kathryn Dovel

**Affiliations:** 1 Department of Global Health, School of Public Health, Boston University, Boston, 02118 MA USA; 2 Partners in Hope, Lilongwe, Malawi; 3 David Geffen School of Medicine, University of California Los Angeles, Los Angeles, CA USA; 4 Department of HIV, Sexually Transmitted Diseases, and Hepatitis, Malawi Ministry of Health, Lilongwe, Malawi

**Keywords:** Differentiated service delivery, HIV treatment, Health systems, Resource-limited settings, stakeholder participation, qualitative research

## Abstract

**Introduction:**

New or returning antiretroviral therapy (ART) clients are largely ineligible for differentiated service delivery (DSD) models. These clients are at increased risk of treatment interruption and may benefit from flexible care models, but stakeholder buy-in may limit progress on interventions for this population. We qualitatively explored stakeholder perceptions and decision-making criteria for scaling DSD models for new or returning ART clients in Malawi.

**Methods:**

We conducted in-depth interviews with internationally-based stakeholders (from foundations, multilateral organizations, and non-governmental organizations (NGOs)) and Malawi-based stakeholders (from the Malawi Ministry of Health and local implementing partners). Interviews included two think-aloud scenarios in which participants rated and described their perceptions of (1) the relative priority of five criteria (cost, effectiveness, acceptability, feasibility, and equity) in determining which interventions to implement for new or returning ART clients and (2) the relative priority of seven potential interventions (monetary incentives, non-monetary incentives, community-based care, ongoing peer/mentor support and counseling, eHealth, facility-based interventions, and multi-month dispensing) for the same population. Interviews were completed in English via video conference and were audio-recorded. Transcriptions were coded using ATLAS.ti version 9. We examined the data using thematic content analysis and explored differences between international and national stakeholders.

**Results:**

We interviewed twenty-two stakeholders between October 2021-March 2022. Thirteen were based internationally and nine were based in Malawi. Both groups prioritized client acceptability, but diverged on other criteria: international stakeholders prioritized effectiveness and Malawi-based stakeholders prioritized cost, feasibility, and sustainability. Both stakeholder groups were most interested in facility-based DSD models such as multi-month dispensing and extended facility hours. Nearly all stakeholders described person-centered care as a critical focus to incorporate into all DSD models.

**Conclusions:**

National and international stakeholders support DSD models for new or returning ART clients. Client acceptability and sustainability should be prioritized to address the concerns of nationally-based stakeholders. Future studies should explore reasons for differences in national and international stakeholders’ priorities and how to ensure that local perspectives are incorporated into funding and programmatic decisions.

## Introduction

Promoting sustained engagement in antiretroviral therapy (ART) services is a major focus of HIV programs throughout sub-Saharan Africa [1]. New or returning ART clients (defined as those on ART < 3 months) are at increased risk of treatment interruption [2–6]. The first few months after (re-)engagement represent a critical period. With targeted interventions, access-related barriers can be reduced and clients can develop the problem-solving skills and external support needed to maintain ongoing engagement in care [7, 8].

Differentiated service delivery models (DSD) are a leading strategy to ease access to lifelong services for stable clients (defined as virally suppressed and/or 6 + months post-ART initiation) [1]. Examples include multi-month dispensing (MMD), community-based ART delivery, and tailored ongoing counseling and peer support. DSD models have a range of benefits, including increased access to care [9], higher acceptability among clients [9, 10],, and improved or non-inferior retention and viral suppression [11–14]. However, new and returning ART clients have largely been excluded from DSD models [15], even though they may stand to benefit most from convenient, private, and lower-cost services [16]. Several DSD models, including incentives [17, 18], peer support [19, 20], community-based ART initiation [13, 14], and e-health interventions [21–23] have been tested among this population in study settings, but are only now being considered for scale and national policy [1].

Stakeholder input can improve intervention design, translation, adoption, and scale-up of successful strategies. However, stakeholder perceptions are not systematically explored, and multi-level stakeholders are often not consulted [24–27]. A deeper understanding of stakeholders’ priorities and constraints can also help researchers offer decision-relevant information, such as data on expected costs and outcomes for different implementation and scale-up scenarios [28, 29].

To our knowledge, no published literature on stakeholder perceptions of DSD models for new or returning ART clients in sub-Saharan Africa exists. We qualitatively explored stakeholder perceptions of DSD models for new or returning ART clients and decision-making criteria for scaling DSD models and compared the views of internationally-based and Malawi-based stakeholders. Malawi was an ideal study setting as it has been the vanguard of innovative public health strategies in the region (such as Option B + and HIV self-testing) despite its highly resource-constrained health system [30–33].

## Methods

### Study design

We conducted in-depth interviews with internationally-based stakeholders (from foundations, multilateral organizations, and non-governmental organizations (NGOs)) and Malawi-based stakeholders (from the Ministry of Health and local implementing partners) to explore perspectives and priorities regarding interventions targeting new or returning ART clients. The study was embedded in the Identifying Efficient Linkage Strategies for Men (IDEaL) randomized controlled trial, which aimed to develop and test the impact of male-tailored differentiated models of care on men’s ART initiation, re-initiation, and early retention in Malawi. The trial is described elsewhere [34].

### Theoretical framework

We used the Assessing Cost-Effectiveness (ACE) approach as our theoretical framework for data collection and analysis [35]. ACE is a structured method for conducting health policy priority-setting studies. It combines rigorous economic evaluation with a qualitative assessment of other implementation factors that influence policy adoption. Stakeholders provide guidance and feedback at every stage of the study process [35].

### Population and recruitment

We aimed to recruit policy and programmatic stakeholders who were experts on DSDs and/or retention in care for new or returning ART clients. We sampled purposively to represent different organizations, including international foundations, multilateral organizations, NGOs, the Malawi Ministry of Health (MoH), and local implementing partners within Malawi. Though clients and community-based organizations are important stakeholders, they were not included in the sample as this study focuses on policy and guidelines decision-making.

Twenty-nine potential participants were identified through internet searches and the research team’s professional networks. Eight additional contacts were added through snowball sampling. Each of the 37 invited stakeholders received two direct outreach attempts by email. We intentionally attempted to sample similar numbers from international and national stakeholder categories to understand the similarities and differences between the two groups.

### Data collection and analysis

We developed an interview guide based on the ACE framework as well as literature on interventions to improve engagement in care among new or returning ART clients in sub-Saharan Africa [36, 37]. The interview guide included open-ended questions about stakeholders’ HIV-related priorities, perceptions of factors influencing non-initiation or attrition immediately following initiation, challenges and solutions for financing and implementing relevant interventions, and desired data to inform decision-making.

Stakeholders also completed two interactive tasks. First, they rated five scale-up decision-making criteria commonly used in the Assessing Cost-Effectiveness (ACE) approach (cost, effectiveness, equity, feasibility, acceptability) as “lower,” “moderate” or “high” priority. We encouraged them to rate no more than three criteria as high priority, and at least one as lower priority. We asked stakeholders to verbalize their thoughts as they completed the task, following the “think-aloud” method,” for qualitative research [38]. After the task was complete, we offered participants the option to rate any additional decision-making criteria and engaged them in retrospective reflection about the activity and the reasons for their ratings.

Following the same format as the previous task, stakeholders rated the relative priority of seven ART initiation and early retention interventions. Options included monetary incentives, non-monetary incentives, community-based care, ongoing peer/mentor support and counseling, eHealth, facility changes, multi-month dispensing, and an “other” category. To construct an average rating of each intervention, we assigned each high-priority rating ten points, each moderate-priority rating 5 points, and each lower-priority rating 0 points [39].

We conducted two pilot interviews to refine and finalize the data collection tool. Two researchers conducted each interview in English via video conference. We recorded the interviews and transcribed them verbatim.

We developed a codebook using a priori codes informed by the existing literature and the theoretical framework [35], as well as inductive codes based on emergent themes [40]. Using Atlas.ti v9 [41], two researchers (KP and KH) piloted the codebook with six interviews. Four researchers (KD, SH, KH, and KP) reviewed the coded transcripts, discussed discrepancies, and refined the codebook. One researcher (KH) coded the remaining data and other researchers performed spot checks on half of the transcripts to ensure consistency. We extracted coded texts and performed thematic content analysis [42]. In the present analysis, we compared and explored differences in themes by international and national stakeholders. We prioritized themes mentioned by many participants and also explored divergent views.

### Ethical considerations

The study was approved by the National Health Science Research Committee (NHSRC) in Malawi and by the University of California Los Angeles Institutional Review Board. All participants gave oral consent before completing an interview. No identifiable information was collected.

## Results

### Participant characteristics

Of the 37 invited stakeholders, 22 agreed to participate. We conducted interviews between October 2021 and March 2022: thirteen with internationally-based stakeholders and nine with nationally-based stakeholders (Table 1). Participants had varied levels of experience with HIV service policies, guidelines, and practice, ranging from 2 to 21 years working in the HIV field.

**Table 1:** Participant characteristics

Characteristic	*N* (%) *N* = 22
Location	
International	13 (59%)
National	9 (41%)
Gender	
Male	15 (68%)
Female	7 (32%)
# of years in current role (Range)	5 (1–20)
Mean # of years working in the HIV field (Range)	14 (2–21)

### Decision-making criteria

**Fig. 1:**
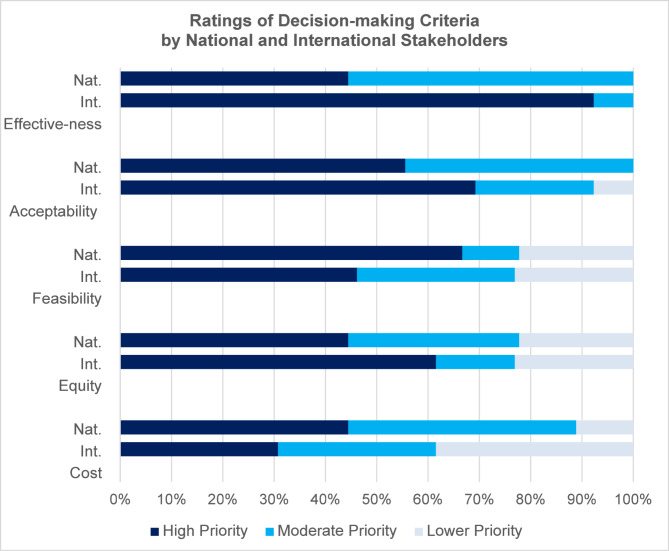
Ratings of decision-making criteria by national and international stakeholders

Overall, intervention effectiveness and acceptability were the highest-priority decision-making criteria (Fig. 1). About half of the stakeholders explained that the decision-making criteria are interrelated, making it difficult to fully distinguish them. For example, a feasible intervention would also be low-cost, and an equitable intervention may have greater long-term effectiveness.

Effectiveness was a high priority for nearly all international stakeholders. International stakeholders often described effectiveness as their first consideration: “There’s no use in implementing something that will not help us reach the expected outcome.” Fewer than half of international stakeholders rated feasibility as a high priority. They explained that implementation challenges can be overcome through innovation, change management, and quality improvement: “Even if an intervention doesn’t seem feasible initially, if you can continually create evidence that… it’s effective, then systems can be built to figure out how that [can] work.”

International stakeholders believed that the costs of interventions are minimal after initial investments because interventions and programs can become more efficient over time: “Just because [something] is expensive today, we might figure out a way to do it more cheaply later”. Some noted that interventions are cost-saving in the long run: “In the long-term, [care] might be less costly if you don’t need to be… spending that much time with people that are coming with advanced disease.”

In contrast, national stakeholders acknowledged the importance of effectiveness but believed feasibility, cost, and sustainability were more important. National stakeholders emphasized the need to select interventions that fit within existing systems and human resource and equipment constraints:

If [an intervention] is not feasible it will not come close to success…it needs to be straightforward and fit well into the rest of the programs and activities that are already present. It needs to fit well with the healthcare workers’ capacities and capabilities. (National Stakeholder)

For a long time, as you know, the whole HIV program like in Malawi is donor dependent, donor-driven… after the project mode has been phased out, it’s very difficult for us as a country to maintain it. So…[it] is necessary to make sure that we have sustainable interventions that can be implemented without donor support. (National Stakeholder)

Nearly all national stakeholders were deeply concerned about the ongoing costs of interventions, citing an expectation that reductions in donor funding are imminent. As one national stakeholder summarized:

It’s easy to find funding for innovations, but very hard to find funding to sustain services. We know that every year the funding for HIV programming goes down… If we’re talking about introducing a certain innovation, are we able to sustain this? When [a donor] is no longer there, this intervention…will die a natural death. (National Stakeholder)

Both international and national stakeholders considered acceptability, especially client acceptability, to be a high priority. As one international stakeholder summarized: “If clients don’t like it, then it’s just going to fail, so it doesn’t matter how cheap and easy it is to do.” Healthcare worker acceptability also was seen as important due to the importance of staff satisfaction and the difficulty of implementing programs without healthcare worker buy-in:

I think some things might not be acceptable to healthcare workers but be super acceptable to people living with HIV or vice versa…but I would advocate that maybe if health care workers don’t love [an intervention] but it is great for communities and people living with HIV, then we’ve got to try [it]. (International Stakeholder)

Some stakeholders discussed the tradeoff between equity and efficiency, explaining that reaching key populations and serving rural areas may be more expensive but is still worthwhile. A couple of national stakeholders prioritized efficiency over equity:

At the end of the day [it comes down to] the number returned into care because that will make it more cost-effective. Yes, I may want to ensure equity by having those extended working hours, but I’m only seeing eight men. And will eight men improve overall retention for [the] country? (National Stakeholder)

### Perspectives on interventions for new or returning ART clients

**Fig. 2:**
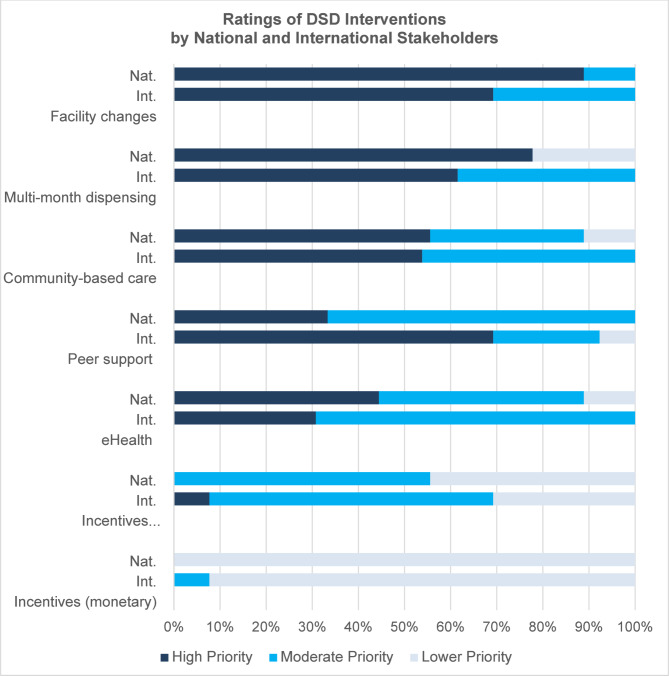
DSD intervention ratings by national and international stakeholders

Overall, international and national stakeholders had similar priorities for DSD interventions (Fig. 2). They noted that multiple models are needed to address the full range of factors causing treatment interruption among new or returning ART clients. International stakeholders were more interested in ongoing peer support/counseling than national stakeholders. The highest-rated interventions were facility changes followed by multi-month dispensing (MMD) (Table 2).

#### Facility efficiencies

Most international and national stakeholders prioritized facility changes (like extending hours of services and reducing wait times) because they saw these changes as feasible within existing systems and infrastructure. They saw healthcare facilities as the foundation of HIV service delivery and central to client experiences of care: “We already have the facilities in place, but what we have not done is try to look at things like the client flow, opening hours. This could be easily done.” (National Stakeholder).

A couple of stakeholders also supported the idea of differentiating facility-based care so that more intensive services are offered to clients with the greatest needs:

We already have workflows that are stripped to the bare bones. The reality is that at most sites, patient consultations only take a few minutes and are not conducted by a trained health worker. There isn’t any room to simplify any further, so the question is what we can add for patients who need it rather than what we can simplify. (National Stakeholder)

#### Multi-month dispensing (MMD)

MMD was also prioritized highly by both international and national stakeholders because it is feasible, acceptable, low-cost, and frees facility resources to support higher-risk clients. Several stakeholders emphasized that new or returning ART clients may require several visits before being given MMD to ensure they are adequately prepared for lifelong treatment, however, many international stakeholders believed MMD should be offered as early as possible:

I assume that people look at the first six months of ART and dread the amount of work it involves…the less they have to do, the less onerous it will seem, and we hope that will make [HIV treatment] feel like something they want to do and want to stick with. (International stakeholder)

#### Peer support and counseling

International stakeholders prioritized peer support highly, emphasizing its effectiveness. In contrast, national stakeholders focused on the high cost and human resource requirements of these programs, though they did support a new MoH program in which a facility-based counselor provides support to clients living with depression, anxiety, and substance use disorder.

Stakeholders believed peer support must be provided in addition to interventions addressing service accessibility: “We use peers a lot… But if you just make it easier to access services then you won’t need all of this additional support” (International stakeholder).

#### Community-based care

Both groups rated community-delivered ART as a moderate priority. Perceived benefits included reduced client time and cost for accessing services and reduced risk of unwanted status disclosure at facilities. Despite these benefits, both international and national stakeholders were concerned about the human resources needed to implement community services. For this reason, several believed that community services should be limited to key populations or those who were especially ill:

It’s very difficult to imagine how this will be done at scale in the national program. We have to remember that this takes health workers away from the facilities [and] it takes many more nurses to see the same number of clients if you send them out in the community. So I think in a health system that is overall understaffed, it is a great luxury to send nurses to meet people in the community. (National Stakeholder)

#### E-health strategies

The majority of stakeholders rated e-Health interventions as a moderate priority. Most stakeholders described e-Health as a new, “untapped” platform that could increase the reach of existing interventions, such as peer support, health education, appointment reminders, SMS check-ins between visits, and tracing clients with missed visits. Yet several believed the technology and evidence were not yet ready for widespread implementation in settings like Malawi.

Stakeholders discussed both equity concerns and benefits related to e-Health interventions. More than half discussed uneven access to phones and the internet as a critical barrier to effective and equitable e-Health interventions, particularly in resource-constrained settings like rural Malawi. National stakeholders noted that individuals often share phones with relatives, creating a risk of unwanted disclosure. However, several stakeholders noted e-Health’s unique potential to connect with harder-to-reach populations such as youth, men, and mobile and rural populations.

#### Incentives

Monetary and non-monetary incentives were the lowest-rated interventions. Stakeholders perceived incentives as highly effective but dismissed the possibility of implementing them because of their cost and complexity. Stakeholders also believed that incentives may reduce clients’ intrinsic motivation for lifelong treatment:

If you tell them, ‘we can give you transport money’ and then we don’t have it, the next time they don’t show up. But if at the beginning you did not tell them that [they would] receive cash, they would find their own means to come to the clinic. [Because of incentives] they develop a dependency syndrome. (National Stakeholder)

Stakeholders did note that targeted incentives can improve equity by supporting poorer clients in meeting daily needs and overcoming socioeconomic barriers to care but were concerned that other clients would find targeted incentives unfair. Some suggested that free or low-cost non-monetary incentives could instead be used to reward or appreciate clients for their engagement in care.

### Overarching service-delivery priority: Person-centered care

Person-centered care (PCC) organically emerged as a high priority for both national and international stakeholders. Stakeholders described PCC as flexible and tailored services that respond to clients’ holistic needs. The three components of PCC discussed most frequently were: (1) segmented care, (2) integrated care, and (3) positive and empowering interactions with healthcare workers.

#### Segmented care

The majority of both international and national stakeholders expressed the view that services should be adapted to meet the needs of diverse groups, such as clients returning to care v. stable clients, men, key populations, clients who prefer private services, and clients with psychosocial needs: “…it’s not a one size fits all approach… what works for female sex workers may not necessarily work for men who have sex with men” (international stakeholder).

A few international stakeholders believed that segmented services may reduce health system costs by limiting costlier interventions to the clients who would benefit from them the most. However, one national stakeholder emphasized that tailoring services for different populations is an “extra project” that is not government-funded and requires additional training and staff time.

#### Integrated services

Integrating HIV services with other healthcare was a priority for both international and national stakeholders, though several national stakeholders believed that integration would be costly and infeasible. Stakeholders described the holistic benefits of integrated care, including reduced costs for clients (because visits are combined), reduced risk of unwanted disclosure of one’s HIV status, and improved overall health outcomes:

They come to the clinic, they get their ART refilled, and tomorrow they’re supposed to go to the clinic to get their diabetes medication or hypertension medication, that to me is a bad idea. [If we had a] one-stop center where you get your ART refills and your other medications, I think that would help. (National Stakeholder)

#### Positive and empowering interactions with healthcare workers

Positive client-healthcare worker interactions, in which healthcare workers are non-judgmental and friendly, were considered key to retaining clients in care and reducing HIV-related stigma. As one national stakeholder stated, interactions with healthcare workers should be “empowering and supportive” rather than “coercive and threatening.” Stakeholders believed that counseling sessions should not be “generic,” but rather tailored to clients’ concerns and designed to foster trust so clients feel comfortable discussing their barriers to treatment:

What I’ve seen from my own experience is that most providers don’t understand that we are all human beings. We have other things that we do apart from coming to the clinic to get medication. So sometimes when a client misses an appointment and comes for a refill, he is treated like he is being punished. [If we] change the attitudes of our providers, I don’t think we are going to struggle with retention or even initiation. (National Stakeholder)

About a quarter of international and national stakeholders noted that the lack of support and education clients receive when initiating ART leads to treatment interruptions:

If there is no time to discuss [treatment barriers] and to encourage disclosing such issues they will of course drop out because they had problems that haven’t been addressed. And I think that can only be addressed if there is… more time for [clients] to fully understand [their diagnosis] and [problem-solve] with treatment supporters and family members. (National Stakeholder)

Nearly all international stakeholders and a couple of national stakeholders emphasized the importance of empowering clients by giving them choices in how they receive care, such as community or facility-based care, peer support, and choice of different facilities. A few stakeholders noted that client needs change over time so explicitly asking about client preferences on an ongoing basis can help identify and meet evolving needs:

[If] as a client I make a choice…whether it is home-based care or whether it is multi-month dispensing, that’s the one that is acceptable for me and therefore I’m likely to adhere to that intervention and have improved linkage and early retention. (National Stakeholder)

**Table 2:** Stakeholder perceptions of interventions

Intervention	Average Rating^†^	Positive Perceptions	Negative Perceptions
Facility efficiencies (e.g. extended hours, improving workflows)	9.0	Acceptable (clients) Feasible Low-cost	
Multi-month dispensing	7.9	Acceptable (clients, policy-makers) Feasible Low-cost	
Community-based care	7.5	Acceptable (clients) Equitable	High-cost Infeasible (complexity + HR requirements)
Ongoing peer support	7.4	Acceptable (clients) Effective	High-cost Infeasible (complexity + HR requirements)
e-Health	6.6	Effective (high future potential) Low-cost	Inequitable (access to phones and the Internet)
Incentives	3.3 (non-monetary) 0.2 (monetary)	Acceptable (clients) Effective (short-term)	High-cost Ineffective (long-term) Infeasible (determining and tracking eligibility)

†Rating scale: High Priority = 10 pts, Moderate-priority = 5 pts, Low Priority = 0 pts

## Discussion

Stakeholders in resource-constrained settings make difficult trade-offs across multiple criteria (such as effectiveness, equity, budgetary and practical constraints, and political considerations) when deciding which interventions to implement and scale up. In this study, we explored how stakeholders make these decisions in the context of interventions for new or returning ART clients in Malawi. Our study suggests that both national and international stakeholders prioritize client acceptability but diverge in other areas: program effectiveness was a higher priority for international stakeholders, while ongoing costs, feasibility, and sustainability were higher priorities for national stakeholders. Despite these differences, international and national stakeholders had similar intervention preferences; they prioritized simple, low-cost, facility-based interventions that remove barriers to care, such as multi-month dispensing and extended facility hours. Most stakeholders attributed their interest in various interventions to PCC, whereby clients are provided tailored services with positive and empowering healthcare worker interactions.

Our analysis suggests notable differences in how international and national stakeholders perceive the long-term costs of interventions. International stakeholders viewed interventions as having low ongoing costs after initial investments. In contrast, national stakeholders were deeply concerned about the long-term costs and resource requirements of interventions. National stakeholders described experiences of watching promising new programs end after donors left. Additionally, international stakeholders believed that factors such as cost and feasibility could be addressed through implementation strategies, a view that was not expressed by national stakeholders.

These findings highlight differences in experiences and perceptions of both global and local history and context. There is a growing consensus that external funding should be aligned with national priorities [43, 44]. However, transitioning from preferred narratives to actual practice may be slow. Historically, key donor funding institutions drive the process of intervention selection and initiatives are managed by numerous NGOs (some locally- and some internationally- based) rather than national governments [45]. International donor funds have predominately supported vertical programs rather than infrastructure or health system strengthening efforts [46]. A locally-driven decision-making process may start with a range of intervention options selected by the Ministry of Health and community stakeholders that are then decided upon jointly. This would help ensure that international and national stakeholders are working together to fund programs that are country-owned, sustainable, and coordinated.

Despite differences in their decision-making criteria, international and national stakeholders had similar intervention priorities, perhaps because of their ongoing discussions. Interestingly, cost and feasibility seemed to have the greatest influence on intervention preferences, though effectiveness and acceptability received the highest ratings in the think-aloud priority-setting tasks in this study. Similar to findings from a previous qualitative study [47], high-level stakeholders favored simple interventions with minimal costs that removed structural barriers to care (e.g. extended hours, MMD) over those that were perceived as highly effective but required additional systems and human resources (e.g. peer support and community-based care).

Stakeholders universally agreed on the importance of PCC, despite their concerns about resource constraints and sustainability challenges. In line with findings from previous PCC studies [48], stakeholders were more interested in PCC’s impact on health system goals (e.g. retention in care, reducing costs) rather than client goals (e.g. living a full life). They viewed PCC as a strategy to reduce healthcare costs by improving the effectiveness and efficiency of programs and recognized the critical importance of client choice and tailored, respectful, holistic care in improving retention and adherence. Additional evidence on the impact of PCC and best practices from sub-Saharan Africa is urgently needed. PCC practices were developed in high-income countries, and little quantitative evidence on PCC in LMICs exists [49]. Some aspects of PCC (such as segmented and integrated care) may require additional resources to be successfully implemented and sustained in historically vertical, disease-specific programs with scarce resources. In such contexts, innovative, low-cost strategies for offering tailored and/or integrated services may be needed. However, recent qualitative research in Malawi suggests that returning male ART clients value positive and empowering relationships with healthcare workers more than where and how ART is delivered, suggesting that key components of PCC could be taken to scale at low cost [50].

Our study has several limitations. First, the think-aloud priority-setting tasks did not fully mimic real-world situations, in which stakeholders consider many nuances of a particular context. Second, there may have been social desirability bias or differences between expressed and revealed preferences. We believe this was minimal as interviewers expressed neutrality and encouragement toward all comments and conversations were frank and casual. Third, not all stakeholders were represented in this study; critical stakeholders such as clients and community advocacy groups were not included due to the study’s focus on policy and guideline decision-making. Fourth, the quantitative results of the two ranking tasks should be interpreted with caution, due to the small sample size. Fifth, some findings may not be generalizable beyond Malawi, particularly concerns about phone access. Despite these limitations, we believe that the priorities and preferences expressed by stakeholders in this study are reflected in real-world settings.

## Conclusions

We found that the top priorities of international and Malawi national stakeholders regarding DSD interventions for new or returning ART clients are effectiveness, feasibility, sustainability, and client acceptability. International stakeholders should recognize and act upon the greater priority national stakeholders place on feasibility and sustainability. Person-centered care was emphasized by all stakeholders and should be incorporated into any intervention for new or returning ART clients. Findings can inform HIV treatment intervention development and research. Further research is needed to understand how differing priorities affect public health discussions, decision-making, and impact, and how to ensure national and local needs are prioritized.

## Data Availability

The data that support the findings of this study are available from the corresponding author, SH, upon reasonable request.

## Abbreviations

ACE: Assessing Cost-Effectiveness
ART: Antiretroviral therapy
DSD: Differentiated service delivery
HIV: Human immunodeficiency virus
MoH: Ministry of Health
MMD: Multi-month dispensing
NGO: Non-governmental organizations
PCC: Person-centered care
